# P12 Improving patient outcomes: a 3 month review of the pharmacist-led COpAT service at University Hospitals of Derby and Burton

**DOI:** 10.1093/jacamr/dlaf239.016

**Published:** 2026-01-05

**Authors:** Anne Boadi, Chris Durojaiye, Charlotte Fiori

**Affiliations:** University Hospitals of Derby and Burton NHS Foundation Trust, Derbyshire, UK; University Hospitals of Derby and Burton NHS Foundation Trust, Derbyshire, UK; University Hospitals of Derby and Burton NHS Foundation Trust, Derbyshire, UK

## Abstract

**Background:**

The Complex Outpatient Antimicrobial Therapy (COpAT) service facilitates the safe delivery of high-risk oral antimicrobial regimens in the community, aiming to optimize clinical outcomes and reduce adverse effects. The definition of ‘complex’ in this context is patient-specific may relate to the antimicrobial regimen, the patient's clinical profile or infection characteristics. Pharmacists play an increasingly integral role in patient monitoring within the COpAT pathway, identifying and managing adverse drug reactions (ADRs) and potential toxicities.

**Objectives:**

To evaluate the clinical impact and effectiveness of the pharmacist-led COpAT service at University Hospitals of Derby and Burton (UHDB) over a 3 month period, focusing on patient outcomes, ADR management, and the nature and scope of pharmacist-led interventions.

**Methods:**

A retrospective review was conducted of all patients referred and enrolled in the COpAT pathway at UHDB between May and July 2025. Specialist COpAT pharmacists assessed suitability for enrolment and monitored for ADRs, drug interactions, abnormal blood results, and adherence. Pharmacists also liaised with multidisciplinary teams (MDTs) as required. Data collection involved a retrospective analysis of pharmacist documentation to identify and categorize clinical issues flagged during monitoring.

**Results:**

Twenty-eight patients (age 36–89 years; 19 male, 9 female) were monitored. Infections included osteomyelitis (*n*=17), spinal infections (*n*=3), prosthetic joint infections (*n*=2), diabetic foot infections without osteomyelitis (*n*=2), septic arthritis (*n*=1), bacteraemia (*n*=1), urinary tract infection (*n*=1) and intra-abdominal abscess (*n*=1). Thirteen patients received oral linezolid and fifteen received oral co-trimoxazole, with several receiving additional agents such as sodium fusidate, ciprofloxacin or metronidazole. Outcomes showed 13 patients (46%) completed therapy without complications, 4 (14%) completed therapy with ADRs, 3 (11%) completed therapy following a treatment change, 6 (21%) failed therapy due to deterioration or intolerance and 2 (7%) had unknown outcomes due to non-compliance. ADRs were agent specific. Among patients receiving linezolid, nausea was most reported (23%), with additional reports of headache (*n*=2), dizziness (*n*=1), metallic taste (*n*=1), sleep disturbance (*n*=1), cracked sore mouth (*n*=1), and bruising (*n*=1). In the co-trimoxazole group, hyperkalaemia affected 20% of patients, with other ADRs including thrombocytopenia (*n*=2), acute kidney injury (*n*=1), neutropenia (*n*=1), and leukopenia (*n*=1). Pharmacists made multiple clinical interventions. Three instances involved additional counselling beyond the initial antibiotic review, supporting ADR management and adherence. Antiemetics were prescribed three times to manage linezolid-associated nausea. Pharmacists escalated abnormal blood results on eight occasions, including thrombocytopenia, hyperkalaemia, leukopenia, acute kidney injury, and low haemoglobin. Four interventions involved coordinating antibiotic regimen changes with MDTs, and in five cases pharmacists liaised with parent teams to escalate clinical deterioration or address complex decisions.

**Conclusions:**

This evaluation highlights the valuable contribution of pharmacists in delivering a safe, effective and patient centred COpAT service. Through proactive monitoring, pharmacists play a pivotal role in managing adverse effects, ensuring adherence, and optimizing clinical outcomes, while also driving antimicrobial stewardship. Embedding pharmacists formally within this pathway enhances stewardship efforts and reduces inpatient burden. Beyond clinical benefits, this model has potential to improve patient experience, reduce healthcare costs, and be scaled across other services to strengthen outpatient antimicrobial care delivery.

